# Oxytocin Receptor Single-Nucleotide Polymorphisms Are Related to Maternal–Infant Co-Occupation and Infant Sensory Processing

**DOI:** 10.3390/children11101196

**Published:** 2024-09-29

**Authors:** Nicki L. Aubuchon-Endsley, Madeline Hudson, Brittany Banh, Emma Opoku, Jason Gibbs, Bryan M. Gee

**Affiliations:** 1 Department of Psychology, Idaho State University, Pocatello, ID 83209, USA; jason.gibbs@nocdhelp.com; 2 Department of Psychology, The University of Tulsa, Tulsa, OK 74104, USA; meh3668@utulsa.edu (M.H.);; 3 Department of Physical and Occupational Therapy, Idaho State University, Pocatello, ID 83209, USA; bryan.gee@rm.edu; 4 Department of Occupational Therapy, Rocky Mountain University, Provo, UT 84606, USA

**Keywords:** maternal–infant co-occupation, infant sensory processing, oxytocin receptor, single-nucleotide polymorphisms

## Abstract

Background: Caregiver–infant reciprocity is related to infant/toddler development and health. However, there is a dearth of research on reciprocity variables like co-occupation and developmental variables such as infant/toddler sensory processing/preferences, and it is important to understand the biopsychosocial mediators of these relations. These include novel genetic markers like maternal oxytocin receptor single-nucleotide polymorphisms (OXTR SNPs). Therefore, this study examined whether mothers carrying risk alleles for three OXTR SNPs displayed different co-occupational behaviors with their infants and whether their infants/toddlers showed different sensory processing/preferences. Methods: Data from the Infant Development and Healthy Outcomes in Mothers Study included prenatal saliva samples assayed for OXTR SNPs, 6-month postnatal behavioral observations coded for maternal–infant co-occupations (reciprocal emotionality, physicality, and intentionality), and 10-, 14-, and 18-month postnatal, maternal-reported Infant/Toddler Sensory Profiles (classified as within or outside the majority range for low registration, sensory seeking, sensory sensitivity, and sensory avoiding). Results: Mothers with rs53576 risk allele A engaged in more frequent reciprocal emotionality, while those with rs2254298 risk allele A engaged in less frequent reciprocal emotionality but more frequent reciprocal intentionality. Mothers with rs53576 risk allele A had infants with 11 times greater odds of being outside of the majority range for sensation avoiding at 10 months old. Conclusions: The results converge with the literature supporting links between OXTR SNPs, caregiver reciprocity, and infant/toddler development but extend the findings to relatively novel constructs (caregiver–infant co-occupations and infant/toddler sensory processing/preferences).

## 1. Introduction

### 1.1. Maternal–Infant Reciprocity

Reciprocity is defined as “joint engagement between infant and caregiver and bidirectional temporal symmetry in actions and psychological states” [1] (p. 1) based on decades of theoretical and empirical work. These interactive, dynamic, and mutually responsive exchanges may include emotions (e.g., positive and negative affect), behaviors (e.g., touch, eye contact/gaze, and movement), and communication (e.g., verbal and non-verbal communications like facial expressions, gestures, and posture) and are highly linked to early attachment [2]. Reciprocity impacts social, emotional, motor, language, and sensory developmental domains and supports key social learning processes that lead to the promotion of core regulatory and cognitive abilities with long-term implications [1,3,4]. Yet, relatively little is known about the infant/toddler sensory processing developmental domain and particular forms of reciprocity, such as reciprocal co-occupation, which has unique implications for theory and practice in occupational therapy.

### 1.2. Sensory Processing

Infant/toddler sensory processing impacts important developmental [5] and health outcomes (e.g., sleep) [6] through salient co-occupations (e.g., breastfeeding) [7]. Sensory processing is defined as our ability to receive, interpret, and respond to environmental stimuli across multiple modalities, which includes the perception and integration of exogenous factors with internal states or cues for appropriate and adaptive meaning making [4,5]. The examination of sensory processing during infancy is salient, given the rate of sensory development during this time and the bidirectional relationships with other domains of development that set the stage for foundational social, executive functioning, and behavioral skills [8]. Individual differences in sensory sensitivity and sensation seeking may arise from problems with the processing and integration of sensory information. This may lead to sensory processing dysfunction that may be present in individuals with autism spectrum disorders [8].

However, empirical research has shown that, even among neurotypical individuals, there is significant variability in important sensory processing domains, consistent with Dunn’s Model of Sensory Processing [9]. Specifically, Dunn’s Four Quadrant Model of Sensory Processing suggests that sensory processing subtypes are derived by thinking about individual differences in two bipolar dimensions based on the constructs of the neurological threshold, or “the threshold for response to a sensory stimulus” [9] (p. 1), and behavioral response, or “whether people have passive or active strategies in response to their environments” [9] (p. 2). Depending on where individuals fall on these two orthogonal axes, they may be high or low in four types of characteristic sensory processing subtypes, including low registration (high neural thresholds, passive responding), sensory seeking (high neural thresholds, active responding), sensory sensitivity (low neural thresholds, passive responding), and sensory avoiding (low neural thresholds, active responding) [9].

Importantly, these sensory processing subtypes have been linked to quality of life in adults, which may predict subjective well-being, functioning across a range of domains, and general health [10]. Additionally, there are direct relationships between sensory processing and mental health, including, but not limited to, common psychological symptoms such as anxiety and depression [11]. Moreover, these links between sensory processing and a broad range of health outcomes have been found across the lifespan, though more research is needed to understand them in infancy [12]. These links may emerge and be significantly shaped by early co-occupations, although there is a dearth of research investigating these associations.

### 1.3. Co-Occupation

Co-occupation is the concurrent engagement of one or more individuals in a shared occupation or activity in which dyadic engagement occurs to reach a common goal [13]. Co-occupation is important given its necessity across contexts (e.g., in family, community, and work settings) and the interdependence of people’s roles across micro-, meso-, and macro-level systems. In fact, collaborative social engagement is an essential process for early learning, bonding, and the successful attainment of developmental milestones [13]. This is why researchers have validated contemporary models of caregiver–infant co-occupation [14] by empirically supporting (1) significant associations between co-occupational subtypes and similar maternal–infant reciprocity constructs (e.g., reciprocal emotionality and caregiver sensitivity to infant affect, reciprocal physicality and caregiver–infant touch, and reciprocal intentionality and caregiver-directed utterances to their infant) [13], (2) the stability of these constructs over the course of infancy and toddlerhood, and (3) expected developmental shifts in caregiver–infant co-occupations due to maturation [15]. The three facets of co-occupation that have emerged are defined as follows: reciprocal emotionality is bidirectional responsiveness to emotional tone, as displayed through emotional cues/expressions and supportive nurturing or comforting behavior; reciprocal physicality is bidirectional physical behavior such as holding or carrying, physical play, or physical redirection; and reciprocal intentionality includes shared purpose, as demonstrated by dyadic co-engagement in essential functions such as feeding/eating, teaching/learning, and shared exploration of the surrounding environment [13,15].

Co-occupational variables may influence infant development through biological risk factors. In particular, maternal genetic risk factors may help to explain relationships between the quality of maternal–infant interactions and both short- and long-term developmental outcomes [16]. In a seminal research article in developmental social neuroscience, Dr. Ruth Feldman details a model of three mechanisms of developmental continuity that explain bidirectional relationships between an infant’s/child’s bio-behavioral regulation abilities and caregiver–infant reciprocity, which together influence developmental outcomes through the infant’s/child’s first 10 years [16]. The model is firmly rooted in contemporary dynamic systems theory and supported by longitudinal research [16]. The first mechanism includes the stability of sensitive parenting, including “the dimensions of reciprocity, mutual responsiveness, or synchrony” [16] (p. 1008), as well as an infant’s/child’s emotion regulation abilities. Such stability leads to repeated experiences that shape and predict long-term behavioral, social, and health outcomes [16]. The second mechanism includes important contextual factors that impact sensitive parenting (e.g., maternal mental health difficulties) and/or offspring emotion regulation (e.g., preterm birth) and thereby significantly influence developmental outcomes [16]. The third mechanism includes “direct, unmediated… biological states or birth experiences… particularly brain stem mediated systems that underpin multiple regulatory functions… or newborn state regulation that shapes the neonate’s orientation to the world” [16] (pp. 1008–1009). The current study hopes to use Feldman’s model as a guiding framework for investigating a specific type of caregiver–infant reciprocity, co-occupation, within the context of important maternal genetic risk markers (see Oxytocin Receptor Single-Nucleotide Polymorphisms Section below) in relation to infant sensory processing, which is a relatively understudied but salient facet of human development.

### 1.4. Oxytocin Receptor Single-Nucleotide Polymorphisms (SNPs)

#### 1.4.1. OXTR SNPs Predict Long-Term Psychological Outcomes

Single-nucleotide polymorphisms (SNPs) are among the most common types of human genetic variations. Oxytocin receptor (OXTR) SNPs are associated with social, emotional, and behavioral outcomes throughout childhood and adolescence (e.g., self-regulation, temperament, empathy, social cognition, and prosocial behavior/skills) [17,18,19]. A recent systematic review [17] also suggests that OXTR SNPs may have a direct relationship with specific facets of autism spectrum disorder, including symptom severity, type, prosocial skills, and indices of neurological functioning. Similarly, OXTR SNPs may interact with early environmental risk factors, including adverse experiences, to predict internalizing symptoms and conduct problems in childhood and adolescence [17], which converges with models such as Feldman’s [16] that highlight the importance of examining gene x environment interactions. These effects may be due to alterations in oxytocin (OT) plasma levels, the regulation of gene expression, and/or oxytocinergic neurons [20].

#### 1.4.2. OXTR SNPs Are Related to Maternal–Infant Interactions

Pursuant to OXTR SNPs’ ability to impact bonding and prosocial behaviors, these genetic mutations are of note to maternal researchers. In particular, maternal–infant interactions have been a key outcome of interest for investigators, focusing on constructs such as maternal sensitivity [21]. Review studies have identified robust associations between OT levels and various markers of maternal–infant interactions and attachment [22]. Further, studies examining the administration of OT to caregivers also suggest that increased OT levels are beneficial for several key outcomes, including parent–child interaction quality, bonding, sensitivity, and synchrony, though these specific impacts seem to be impacted by peripheral variables (e.g., attachment style and the presence of others) [23].

Studies examining various OXTR SNPs have also demonstrated key relationships with maternal parenting variables. One study showed that parents who were homozygous for the risk alleles of OXTR rs2254298 and rs1042778 had lower plasma OT than carriers of the alternative allele. These lower OT levels, combined with the OXTR risk alleles, were associated with fewer instances of parental touch [24]. Conversely, parents with higher OT levels showed longer durations of gaze synchrony with their infants and increased parental care [24]. Similarly, a separate study found that mothers who were homozygous for the risk allele on rs1042778 (TT) showed diminished behavioral sensitivity and lower engagement during a mother–infant teaching task [25]. It was also found that maternal trauma history interacted with genotype, resulting in increased frightened/frightening behavior among TT homozygotes [25]. This supports the notion that the OXTR genotype interacts with other outside variables to impact maternal behavior. Additionally, the OXTR genotype may impact maternal behavior through alterations to related systems. In fact, the findings from one study suggest that OXTR rs2254798 is related to executive functioning, which eventually impacts maternal behavior [26]. OXTR SNPs, such as rs53576, have also been related to maternal sensitivity. Specifically, SNP carriers of the risk (A) allele showed less sensitivity to their toddlers during a puzzle-solving task. This effect persisted even after controlling for maternal education, depression, and marital issues [27].

### 1.5. Current Study

Taken together, these maternal genetic risk markers may at least partially account for associations between maternal–infant interaction synchrony and infant developmental outcomes. However, no known research has investigated relationships among the maternal–infant reciprocity variable co-occupation, OXTR SNPs, and the relatively understudied infant developmental domain sensory processing. While the current study initially aimed to examine mediation models with maternal–infant co-occupation as the primary predictor, maternal OXTR SNP as the mediator, and sensory processing as the outcome, a priori power analyses suggested that these analyses would be underpowered based on available data (*n* < 60). Therefore, the researchers in this study investigated univariate relationships between OXTR SNPs and either maternal–infant co-occupation or infant sensory processing variables in order to understand which specific variables should be examined in future research as well as to lay the groundwork for future mediation models. Based on the existing literature, we hypothesized that mothers with the risk alleles for OXTR SNPs rs53576 (A allele), rs2254298 (A allele), and rs1042778 (T allele) would engage in less maternal–infant co-occupational behavior (assessed via frequency and mean duration of reciprocal emotionality, physicality, and intentionality) and would have infants/toddlers outside of the majority range in each sensory processing domain utilizing Dunn’s Model of Sensory Processing (i.e., low registration, sensory seeking, sensory sensitivity, and sensory avoiding) [9].

## 2. Materials and Methods

### 2.1. Participants

The data were from the Infant Development and Healthy Outcomes in Mothers Study (IDAHO Mom Study; Human Subjects Committee approved protocol 4191), a longitudinal study conducted from 2015 to 2018. Participants were recruited from Southeastern Idaho from a variety of maternal health practitioners, regional professionals, organizations serving families, and social and other media. To be included in the study, pregnant participants had to be between 33 and 37 weeks’ gestation at their first visit and 18–35 years old. Maternal participants were excluded if they reported having major medical (e.g., pre-eclampsia, toxemia, or HIV-positive diagnosis) or mental health difficulties (e.g., bipolar I disorder or schizophrenia) or engaged in excessive substance use or use of medications known to have adverse fetal effects (i.e., Food and Drug Administration categories D or X) during pregnancy. Additionally, participants had to be fluent in written and spoken English to complete informed consent and could not be carrying multiples for the current pregnancy. Infant/toddler participants were excluded if they were at least 2 weeks outside of the required age window. Participant sessions occurred at five time points, including prenatal (33–37 weeks’ gestation; *n* = 125), 6 months postpartum (±2 weeks; *n* = 96), 10 months postpartum (±2 weeks; *n* = 44), 13–14 months postpartum (±2 weeks; *n* = 53), and 18 months postpartum (±2 weeks; *n* = 54), and participants were given USD 30 for each completed session. Participants could continue participating in postnatal sessions regardless of whether they missed one or more prior postnatal sessions [6].

### 2.2. Procedure

Within the IDAHO Mom Study, researchers utilized various measures to assess maternal health and infant development in a rural health provider shortage area. For the current study, OXTR SNPs were analyzed after extracting genetic information from prenatal maternal saliva samples. Maternal–infant co-occupation was coded during standardized behavioral lab tasks at 6 months postpartum, and sensory processing profiles were gathered via maternal reports on the Infant/Toddler Sensory Profile 2 at 10-, 14-, and 18-month sessions. Participants’ saliva was collected using the passive drool method at home following the prenatal session. This method was employed due to its non-invasive nature and decreased risk of sample contamination [28]. Diurnal saliva collection occurred at four time points (i.e., upon awakening, 30 min post-awakening, 45 min post-awakening, and prior to bed at night) across 3 days to capture diurnal changes in salivary cortisol levels despite potential contamination of one or more individual samples [29]. To enhance sample quality, participants were instructed not to eat, drink anything other than water, smoke, or brush their teeth prior to providing samples. After all samples had been collected, research assistants safely transported the samples to freezers within the research lab. Samples were stored at −20 °C and sent to the Idaho State University Molecular Research Core Facility for assaying. Participants were given USD 5 for each day that they completed all four samples.

At the 6-month visit, participants engaged in a series of standardized tasks based on the Laboratory Temperament Assessment Battery [30], including caregiving, free play, orientation, and limitations tasks, with their infants within a structured lab setting to control for potential extraneous influences on maternal or infant behavior [31]. The task was discontinued if the infant displayed an excessive level of distress (defined as red, flushed face with continual crying) for 30 s or longer. At the 10- to 18-month sessions, participants completed paper-and-pencil Infant/Toddler Sensory Profile 2 forms following the series of standardized maternal–infant behavioral tasks.

### 2.3. Measures

#### 2.3.1. Oxytocin Receptor Single-Nucleotide Polymorphisms

The Gentra Puregene Blood Kit (QIAGEN, Venlo, The Netherlands) was used to isolate genomic DNA from viable saliva samples using QIAGEN DNA Purification from Body Fluid. Then, the samples were quantified by a Qubit Fluorometer (Thermo Fisher Scientific Inc., Waltham, MA, USA) [32,33]. DNA from these samples was extracted, purified, amplified, and assayed for the OXTR SNPs rs53576, rs2254298, and rs1042778 using TaqMan SNP Genotyping Assays. The Bio-Rad CFX96 qPCR machine (Bio-Rad Laboratories, Inc., Hercules, CA, USA) was used to assess allelic discrimination [34]. For this purpose, this device used endpoint fluorescence detection. Once identified, the fluorophore was used to identify and differentiate between major and minor alleles.

#### 2.3.2. Maternal–Infant Co-Occupation

Maternal–infant co-occupation variables were continuously coded at the 6-month visit, including the frequency and mean duration of reciprocal emotionality, intentionality, and physicality [13,14,15] from the 7 min free-play tasks. Examples of each type of co-occupational behavior can be seen in Table 1, which was replicated with the authors’ permission [15] and adapted from the prior literature [14].

Two research assistants from the University of Tulsa Psychology Department and one research assistant from the graduate occupational therapy program at Idaho State University completed training sessions with Dr. Bryan Gee to effectively utilize an open-source video analysis software called Datavyu (version v:1.5.3) for behavioral coding. These research assistants also met with Dr. Gee several times to review the definition and behavioral manifestations of each co-occupation and practice with video vignettes. Intra- and inter-rater reliability were calculated by comparing research assistant coding attempt results to a standard created by Dr. Gee. Students had to obtain reliability coefficients of at least 0.80 before their coding data could be used. Co-occupation intra- and inter-rater reliability ranged from κ = 0.85 to 0.93.

#### 2.3.3. Infant Sensory Processing

The Infant/Toddler Sensory Profile 2 (ITSP-2) can be used from birth to 3 years of age to identify strengths and barriers regarding infant/toddler patterns of sensory processing. The mothers in the current study completed the ITSP-2, which includes 36 items for infants between birth and 6 months of age or 48 items for those between 7 and 36 months of age. Items include descriptions of the infant’s/toddler’s responses to everyday sensory experiences [35]. Mothers rate the frequency of these sensory experiences on a 5-point Likert scale. The resulting scores are contextualized using majority ranges, which are the range of scores attained by the majority of similarly aged infants in the standardization sample for each sensory pattern (i.e., Seeker, Avoider, Sensor, and Registration/Bystander) [35]. Dunn defines sensation-seeking items as measuring infants’/toddlers’ interest in sensations and resulting pleasure (e.g., “My child enjoys looking at his/her own reflection in the mirror”) [35]. Sensation avoiding includes items measuring infants’/toddlers’ need to control how much and which type of sensations they experience (e.g., “My child avoids getting his/her face wiped”) [35]. Sensory sensitivity includes items measuring infants’/toddlers’ ability to notice sensations (e.g., “My child is distracted and/or has difficulty eating in noisy environments”) [35]. Low registration includes items measuring infants’/toddlers’ awareness of available sensations (e.g., “My child seems unaware of wet or dirty diapers”) [35].

The ITSP-2 was standardized using samples of infants/toddlers without disabilities from birth to 6 months (*n* = 100) and 7 to 36 months (*n* = 489). In the current study, infants/toddlers were classified as outside of the majority range if their scores were greater than 1 standard deviation above the mean or less than 1 standard deviation below the mean, corresponding to one of the following categories: “much less than others”, “less than others”, “more than others”, or “much more than others” for each quadrant [35]. It is important to note that the ITSP-2 is used to identify sensory preferences and not sensory disabilities or disorders. Scoring in the “more than others” or “less than others” ranges does not necessarily indicate a functional problem, but these categories were included as outside of the majority range for the current study given the community sample.

Higher and lower scores in each quadrant correspond to unique potential barriers to fully engaging with the surrounding environment. For example, those with sensation seeking lower than others (less or much less) may not seek input and show less interest and motivation to engage in sensory activities, while those with sensation seeking higher than others (more or much more) may seek input excessively, which reduces their ability to participate in their environment. Those with sensory avoiding lower than others (less or much less) may have difficulty limiting sensory input despite overstimulation, while those with sensory avoiding higher than others (more or much more) may be so overwhelmed by sensory input that they are unable to fully engage with the environment. Those with sensory sensitivity lower than others (less or much less) may fail to detect important details, while those with sensory sensitivity higher than others (more or much more) may be so distracted that they are unable to fully participate in their environment. Those with sensory registration lower than others (less or much less) may notice sensory input that is not helpful for participation in their environment, while those with sensory registration higher than others (more or much more) may miss important sensory information.

The results from a recent review of the Sensory Profile-2 using the Consensus-based Standards for the Selection of Health Measurement Instruments and Quality Criteria for Health Status Questionnaires provide evidence that the measure demonstrates adequate to good internal consistency, content validity, and construct validity [36]. Additional studies have supported good convergent validity between the Child Sensory Profile 2 and Sensory Processing Measure [37]. Likewise, a systematic review of sensory processing assessments suggests that the ITSP-2 has excellent content validity based on an expert panel review, adequate to good construct validity when compared to the Infant/Toddler Symptom Checklist, adequate to good test–retest reliability for those 7 to 36 months of age (quadrant scores *r* = 0.74), and adequate internal consistency for those 7 to 36 months of age (quadrant scores *α* = 0.70–0.86) [38], suggesting that it is a psychometrically sound measure for the current study sample.

### 2.4. Quantitative Analyses

Point-biserial correlations were used to examine allele-group differences in co-occupational variables. In particular, we examined whether there were differences in the frequency and mean duration of each co-occupational variable (i.e., reciprocal emotionality, physicality, and intentionality) between mothers who have the risk allele for each OXTR SNP and mothers who do not. Chi-square tests of independence were used to examine relationships between allele groups and infant sensory processing. In particular, we examined whether there was a relationship between mothers having versus not having the risk allele for each OXTR SNP and infants being within versus outside of the majority group for each sensory processing quadrant (i.e., low registration, sensory seeking, sensory sensitivity, and sensory avoiding).

## 3. Results

### 3.1. Descriptive Statistics

To understand the current sample for contextualization, replication, and generalization purposes, information is provided here regarding key descriptive information about mothers and infants/toddlers (see [6,39] for descriptive statistics tables and Consolidated Standards of Reporting Trials [CONSORT] diagrams). During the prenatal visit, sociodemographic information was collected and revealed that the majority of women who enrolled and completed the prenatal visit identified as White/Caucasian (≈88%), married (≈84%), employed (≈60%), and members of the Church of Jesus Christ of Latter-Day Saints (≈62%), with about half (≈49%) attaining a college or university degree and a modal family annual gross income between USD 50,000 to USD 74, 999 (≈29%). However, there was significant variability in combined annual gross income in the sample (range = <USD 5000 to ≥USD 100,000). Most mothers (≈59%) had at least one additional child at home. At the 6-month session, mothers reported infants’ sex assigned at birth and gestational age. About half of the infants were female (≈47%) and half were male (≈53%), and the mean gestational age was considered full-term (≈39.5 weeks) [29]. Based on the maternal-reported date of birth and participants’ dates of postnatal sessions, mean ages were computed at 6- (≈6.1 months), 10- (≈10.1 months), 13–14- (≈14.1 months), and 18-month (≈18.1 months) visits. In addition, the mean and standard deviation values are presented for each 6-month co-occupational variable in Table 2.

### 3.2. Co-Occupation and SNPs

There were differences in the frequency of reciprocal emotionality (rs53576, rs2254298) and frequency of reciprocal intentionality (rs2254298) based upon the maternal allelic configuration (see Table 3). Specifically, mothers with the rs53576 risk allele (A) demonstrated more frequent reciprocal emotionality with their 6-month-old infants. Mothers with the rs2254298 risk allele (A) demonstrated less frequent reciprocal emotionality but more frequent reciprocal intentionality with their 6-month-old infants.

### 3.3. SNPs and Sensory Processing

There were different proportions of infants outside the majority range of sensation avoiding at 10 months of age based on the allelic configuration (rs53576, χ^2^(1) = 4.22, *p* = 0.040, Phi = 0.395). The odd’s ratio of 11.0 suggests that mothers with the risk allele (AA or GA) had infants who had 11 times greater odds of being outside of the majority range of sensation avoiding at 10 months of age compared to those with mothers who had the GG allelic configuration for this SNP.

## 4. Discussion

### 4.1. Risk Alleles and Co-Occupational Behavior

While there were some statistically significant differences in co-occupational variables based on maternal allelic configuration, contrary to a priori hypotheses, several of these differences were in the opposite direction. Specifically, based on prior published results, we predicted that carriers of risk alleles for each of the OXTR SNPs (rs53576, rs2254298, and rs1042778) would engage in less frequent and briefer co-occupational behavior, which was the case for mothers with the rs2254298 risk allele (A), and less frequent reciprocal emotionality. Nevertheless, the opposite was true regarding the rs53576 risk allele and reciprocal emotionality and the rs2254298 risk allele and reciprocal intentionality. These findings may be due to the fact that the current study was only able to focus on maternal OXTR SNPs without considering infant SNPs. In other words, it may be that the allelic composition/compatibility of both mother and infant OXTR SNPs predicts dyadic behavior more robustly and reliably, which should be investigated further.

Additionally, it may be that genotype information alone is not sufficient to understand how the expression of given genes may impact maternal–infant behavior. Specifically, studies have found that early life experiences such as childhood maltreatment interact with the OXTR SNP genotype (i.e., rs53576) to predict OXTR DNA methylation, which influences oxytocinergic signaling [40]. Thus, epigenetic processes, which are impacted by interacting genetic and environmental factors, may also play a key role in predicting behavior. Likewise, empirical findings suggest that the mean methylation of OXTR between mothers and infants is significantly correlated under certain conditions (e.g., in mothers who did not experience childhood maltreatment) [40]. This means that the degree to which maternal–infant gene expression is related also depends on key facets of mothers’ experiences during sensitive time periods. Therefore, future studies should consider associations between maternal and infant genetic and epigenetic factors, a broader range of markers or indicators of OXTR functioning (e.g., more SNPs or mean methylation), and important environmental factors that are associated with both.

Relatedly, important moderating and mediating variables may help to explain or modify direct associations between maternal SNPs and dyadic engagement and/or attunement [41] (e.g., maternal mental or physical health difficulties, infant care arrangements and quality, birth complications, breastfeeding, cultural influences on parenting or community support, infant mental or physical health difficulties, and socioeconomic resources) and thus should be considered in future studies with sample sizes sufficient for covariates and/or more complex models that can account for these variables.

### 4.2. Risk Alleles and Sensory Processing/Preferences

Similarly, our second set of hypotheses was partially supported in that the rs53576 risk allele was associated with being outside of the majority range of sensation avoiding at 10 months of age. This means that mothers who have the risk allele have infants at greater risk of overstimulation, which may disrupt their ability to optimally engage with and participate in their surrounding environment, which is necessary for typical development, particularly during early, sensitive/critical periods. Interestingly, none of the other SNPs or forms of sensory processing were related at any other time point. This may be due to limited statistical power, but there may also be relatively more robust associations with this particular OXTR SNP and sensation avoiding, which may be more readily observable at the 10-month developmental time period. Notably, there may be direct associations between the caregiver’s and infant’s OXTR SNP allelic profiles, which may place infants at increased risk for sensory processing difficulties. For example, those with the OXTR SNP rs53576 risk allele may have altered processing of particular sensory information (e.g., social auditory processing) [42]. However, it is important to note that relationships between OXTR SNP variants and behavior across generations are complex with multifactorial influences that need to be further investigated.

### 4.3. Potential Explanations for Findings

Preliminary, correlational research also suggests that those with the rs53576 risk allele may have a lower hypothalamic volume in a graded manner based on the allelic profile (i.e., A/A < G/A < G/G) [43]. In the same study, this allele-load dependence was also seen regarding the risk allele and decreased amygdala activation in response to the processing of facial emotions, but there was an increased functional correlation between the hypothalamus and amygdala [43]. Based on these differences in indicators of neuroanatomical structure and connectivity, the authors “speculate that different genetic variants translate into different neural risk mechanisms relating to hyperactive social threat signaling (and possible avoidance behavior) and hypoactive limbic signaling (and possible impairment in social stimulus discrimination and processing)” [43] (p. 13939). Given the roles that these oxytocinergic regions play in sensory processing and integration [44], these neural substrates may also provide an explanation for differences in sensory processing and avoidance in the current sample, though these hypothesized mechanisms are highly tentative, and much more work is needed to understand the biopsychosocial processes that underlie the current study findings.

Prior study results also support an indirect relationship between the parental OXTR SNP rs53576 risk allele and offspring sensory sensitivity through parenting. In particular, researchers have highlighted that carriers of the rs53576 risk allele may be less empathic and engage in less sensitive parenting practices [45]. Additionally, the quality of parenting may serve as a buffer to associations between stronger emotional stimuli and greater arousal [46]. Thus, if infants are more sensitive to exogenous stimuli, potentially based on genetic risk factors, they may have to actively avoid these stimuli when caregivers do not engage in sensitive parenting practices to help lower their infants’ arousal (e.g., soothing, distraction, or minimization of strong sensory stimuli).

### 4.4. Important Developmental Timing

Moreover, parents may begin to observe sensory processing issues as their infants transition to toddlerhood and are able to communicate their sensory aversions more effectively (e.g., trying to remove clothing, intentionally removing themselves from environments with more intense light or sound, or refusing to eat certain foods). This may explain why these findings were statistically significant with 10-month-old infants and suggests that it may be helpful to examine early sensory processing preferences during the important transition from late infancy to early toddlerhood. Overall, previous empirical work supports a gene–environment interaction between OXTR SNPs and parent–child interactions in predicting salient infant outcomes, and it may be particularly helpful to observe these outcomes during important developmental transitions, such as the shift from infancy to toddlerhood [47].

### 4.5. Limitations and Future Directions

Several limitations of the current study should be considered when interpreting its results and can inform future research. For instance, the current study sample was relatively small and homogeneous with regard to sociodemographic and cultural factors. This may limit the generalizability of the current study findings and suggests a need for the current study hypotheses to be tested in larger, more diverse samples of caregivers and their infants/toddlers. Additionally, within the current low-risk community sample, there was significant homogeneity with regard to sensory processing preferences. In particular, from 10 to 18 months of age, most infants/toddlers were within the majority range for each sensory processing quadrant (range = 66–92%; see Table 4). Therefore, future studies may want to utilize recruitment procedures that would allow for a broader representation of infants’/toddlers’ sensory processing preferences and abilities, including enrolling people who are neurodiverse and who may be more prevalent in clinical settings.

Similarly, although 50% of mothers carry the risk allele (A) for rs53576, only 26.3% carry the risk allele (A) for rs225429, and 65.3% carry the risk allele (T) for rs104277. Based on Allele Frequency Aggregator (ALFA) data using the National Center for Biotechnology Information (NCBI) Single-Nucleotide Polymorphism Database (dbSNP) [48], 32.6% carry the risk allele for rs53576, 24.8% carry the risk allele for rs225429, and 38.6% carry the risk allele for rs104277. Therefore, our sample had a similar percentage of risk allele carriers for rs225429 in comparison to population estimates but had a greater percentage of risk allele carriers for rs53576 and a notably greater percentage of risk allele carriers for rs104277. Thus, our sample percentages may be notably disparate from existing population estimates, and significant over- or under-representation of allelic profiles within the sample for any given OXTR SNP may lead to unequal subsample size comparisons. Significant differences between allele proportions between a sample and population and notably unequal subsample sizes both may lead to reduced external validity. The latter also may be problematic for statistical power and may increase the risk of violating homogeneity-of-variance assumptions for certain statistical tests. Therefore, future studies should take both issues into consideration when planning for their sample’s representation of allelic configurations. In addition, enhancing the ethnoracial heterogeneity of the sample may also enhance OXTR SNP genetic variation, which should be a goal for future studies. Follow-up studies should also consider the examination of risk profiles considering multiple SNPs simultaneously as opposed to the examination of individual univariate relationships, which will be more feasible with larger, more diverse samples. Moreover, there may be important interactions between maternal and infant OXTR SNP genotypes, and investigating them in future studies may allow for a more nuanced understanding of relationships between important facets of maternal–infant reciprocity and infant/toddler development.

### 4.6. Conclusions

The current study supports differences in maternal–infant co-occupational behavior at 6 months postpartum based on the OXTR SNP genotype. This includes associations between rs53576 allele A and more frequent reciprocal emotionality and rs2254298 allele A and less frequent reciprocal emotionality but more frequent reciprocal intentionality. In addition, 10-month-old infants with mothers who have the rs53576 risk allele have 11 times greater odds of being outside of the majority range for sensation avoiding. Taken together, these findings further support complex relations between OXTR SNPs and maternal–infant reciprocity and extend the literature to important co-occupation facets while preliminarily supporting associations between OXTR SNPs and infant sensory processing/preferences. More studies need to examine these relations in larger, more diverse samples to examine whether allelic configurations or profiles mediate or moderate relations between maternal–infant co-occupational behavior and infant sensory processing/preferences. Differences were not always in the predicted direction, which may be due to a need to simultaneously consider maternal and infant genetic and epigenetic factors, their comparability, and important early moderators and mediators to these relationships.

## Figures and Tables

**Table 1:** Coding information for co-occupation variables.

Category	Label	Definition *	Behavioral Examples *
Physicality	phy	Reciprocal motor behavior shared between infant and caregiver	Positive or negative physical contact: holding, grooming, bathing, rocking, snuggling, physical redirection, physical play, feeding, breastfeeding, dressing/undressing, dancing
Emotionality	emo	Reciprocal responsiveness to another’s emotional tone	Emotional action that produces reciprocal action: play, soothing, nurturing, comforting, communication, vocal expression
Intentionality	int	Understanding of shared purpose and role of one another during engagement in co-occupation	Feeding, teaching a new skill, redirection, play, exploring

* Adapted from Pickens and Pizur-Barnekow (2009) [14].

**Table 2:** Descriptive statistics for each 6-month co-occupation variable.

Co-Occupational Variable	Mean 6-Month Frequency or Duration	Standard Deviation
Emotionality Frequency	7.22 times	4.43 times
Physicality Frequency	6.62 times	4.16 times
Intentionality Frequency	6.10 times	3.17 times
Emotionality Duration	8.93 s	9.90 s
Physicality Duration	14.77 s	3.21 s
Intentionality Duration	15.75 s	12.55 s

**Table 3:** Point-biserial correlations between allele groups for oxytocin receptor single-nucleotide polymorphisms and co-occupational variables at 6 months of age.

		OXTR SNP		
Co-Occupation Variable	Statistic	rs53576	rs2254298	rs1042778
Emotionality Frequency	*r_pb_*	−0.419 **	0.322 *	−0.109
	*n*	46	45	45
Emotionality Mean Duration	*r_pb_*	0.149	0.130	0.181
	*n*	46	45	45
Physicality Frequency	*r_pb_*	−0.046	0.086	0.002
	*n*	48	47	47
Physicality Mean Duration	*r_pb_*	0.064	0.241	0.248
	*n*	48	47	47
Intentionality Frequency	*r_pb_*	0.148	−0.366 *	0.022
	*n*	47	46	46
Intentionality Mean Duration	*r_pb_*	−0.102	0.161	0.033
	*n*	47	46	46

* *p* < 0.05, ** *p* < 0.01.

**Table 4:** Percentage of infants/toddlers who were above, like, or below the majority range.

	Sensory Processing Domain (Age in Months)			
Sensory Processing Category (10)	Seeker Groups	Avoiding Groups	Sensitivity Groups	Registration Groups
Much Less/Less than Majority	4.4	15.9	6.8	14.3
Like the Majority	75.6	79.5	65.9	78.6
Much More/More than Majority	20.0	4.5	27.3	7.1
Sensory Processing Category (14)	Seeker Groups	Avoiding Groups	Sensitivity Groups	Registration Groups
Much Less/Less than Majority	7.7	5.8	3.8	6.0
Like the Majority	71.2	84.6	75.0	92.0
Much More/More than Majority	21.2	9.6	21.2	2.0
Sensory Processing Category (18)	Seeker Groups	Avoiding Groups	Sensitivity Groups	Registration Groups
Much Less/Less than Majority	9.3	9.3	3.8	9.4
Like the Majority	70.4	75.9	79.2	84.9
Much More/More than Majority	20.4	14.8	17.0	5.7

## Data Availability

The data presented in this study may be available on request from the corresponding author. The data are not publicly available due to specific ethical and privacy considerations.
